# Use of transanal minimally invasive surgery for endoscopic resection of rectal tumour: a technical note

**DOI:** 10.1093/gastro/gou039

**Published:** 2014-07-03

**Authors:** Muhammad Shafique Sajid, Muhammad I. Bhatti, MK Baig, William F.A. Miles

**Affiliations:** ^1^Department of General, Laparoscopic and Endoscopic Colorectal Surgery, Western Sussex Hospitals NHS Trust, Worthing Hospital, UK and; ^2^Department of General Surgery, Queen Elizabeth Hospital, King’s Lynn NHS Foundation Trust, UK

**Keywords:** Rectal cancer, endoscopic transanal resection of tumour (ETART), transanal minimally invasive surgery (TAMIS)

## Abstract

**Background:** The aim of this article is to report and discuss a case of lower rectal cancer undergoing endoscopic transanal resection of tumour (ETART) using a transanal minimally invasive surgery (TAMIS) approach.

**Methods:** A technical note on a case report. An innovative approach for ETART using TAMIS.

**Results:** This is the first-ever case report of lower rectal cancer treated by ETART using a TAMIS approach. The procedure was completed successfully without any operative or peri-operative complication. Peri-operative flexible sigmoidoscopy confirmed a wide and patent rectal lumen.

**Conclusion:** Use of a TAMIS approach for ETART to remove lower rectal cancer for palliation can be technically very effective compared with conventional ETART, due to the potential advantages of avoiding contaminant fluid spillage, easy access, better visualization compared with conventional ETART, and being user-friendly. The results from larger cohorts of patients undergoing TAMIS ETART are required before recommending the routine use of this technique. However, until then, this approach may be considered as an alternative to conventional ETART.

## INTRODUCTION

Due to the mortality and morbidity associated with radical resection of the rectum for large adenomas and early rectal cancer (T1 and T2), the use of local, minimally invasive techniques, such as transanal excision, transanal endoscopic microsurgery, endoscopic mucosal resection, submucosal dissection and transanal minimally invasive surgery (TAMIS), are gaining popularity, equally in the surgical fraternity and among colorectal patients [1]. TAMIS, a relatively innovative modality, facilitates excision of lesions not otherwise amenable to standard transanal excision, thereby extending its utility for polyps of the middle and upper rectum. TAMIS provides enhanced visualization and precise excision, leading to shorter hospital stay and low morbidity and mortality [2]. Numerous barriers have been identified into the widespread adoption of TAMIS such as the need for specialized instruments, associated higher costs and steep learning curve of the technique. Despite this the authors advanced this approach to the next level by using the same method for endoscopic transanal resection of tumour (ETART) in the rectum. ETART is usually performed using urology resectoscopes [3], and is a local approach for either the palliation or the definitive treatment of rectal neoplasms or symptomatic control of anastomotic strictures without the risks of major surgery. The technique of ETART is particularly useful when the lesion lies below the peritoneal reﬂections but not within reach of digital rectal examination. The objective of this article is to report a first-ever case of rectal cancer requiring palliative ETART, which was performed using a TAMIS approach.

## CASE PRESENTATION

**Figure 1:**
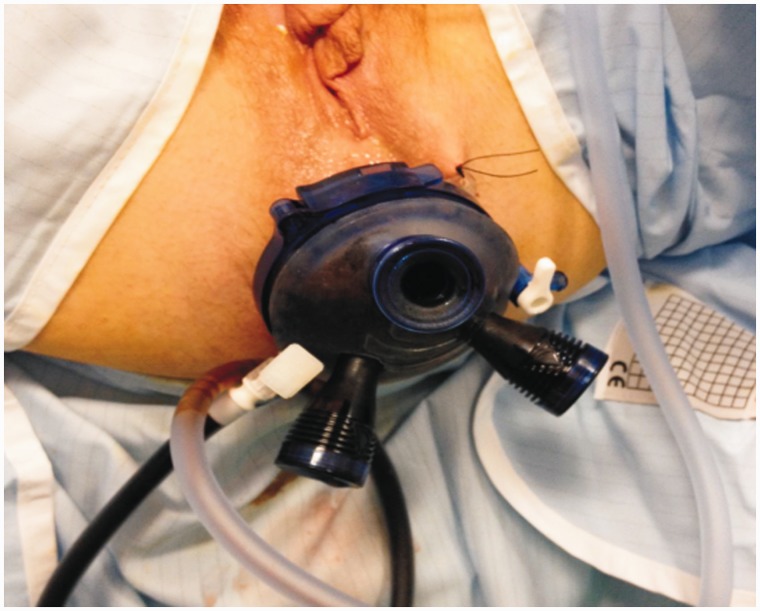
TAMIS port (with three small ports) *in situ* prior to the start of ETART.

**Figure 2:**
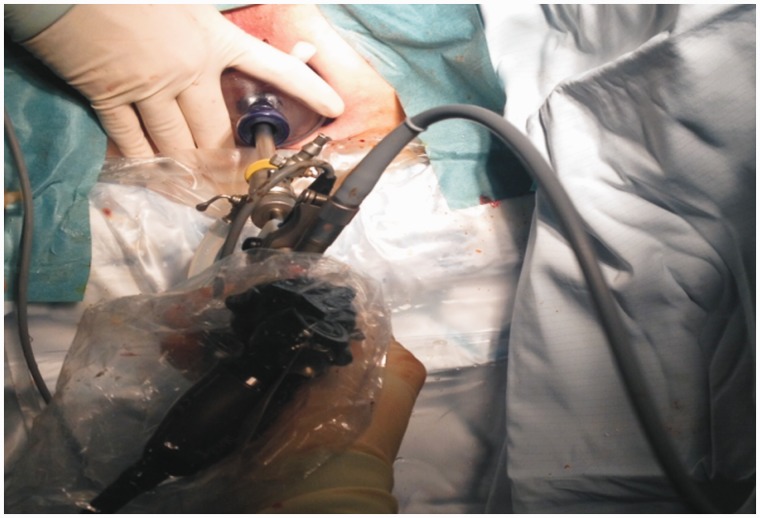
Use of urology resectoscopes via TAMIS port for ETART.

An 83-year-old male presented to the colorectal clinic in May 2010 with history of loose motions and fresh bleeding *per rectum*. He underwent colonoscopy, which revealed a malignant-looking tumour at 12 cm from the anal verge. Histopathology of biopsy from the tumour confirmed adenocarcinoma. This case was discussed at the local colorectal cancer multidisciplinary weekly meeting and it was decided not to offer the patient radical resection in the form of anterior resection, due to associated co-morbidity of a recent cerebrovascular accident that led to hemiplegia and required percutaneous endoscopic gastrostomy for feeding, permanent suprapubic catheter and regular speech therapy. His other comorbidities included hypertension, benign prostatic hypertrophy and gastro-oesophageal reflux disease. On developing signs and symptoms of large bowel obstruction, the patient had undergone standard ETART with a urology resectoscope twice in the previous four years. Recently, he presented with another episode of large bowel obstruction, attributed to a large rectal tumour. He underwent flexible sigmoidoscopy, which revealed an impassable tumour in the lower rectum. Due to difficulties over access, poor visualization, and soiling caused by water leakage with standard ETART instruments, the authors employed the TAMIS approach for ETART (Figures 1 and 2). A multiple access port (GelPort®; Applied Medical, London, UK) was used for endo-anal access, which dramatically improved visualization and secured a watertight device–anal placement. One port was utilized for camera access and the remaining two were used for the resectoscope, suction irrigation and various other manoeuvres. The procedure involved removing small chunks of tumour tissue with each sweeping movement of the resectoscope, under direct vision. Each quadrant of the tumour region was removed systematically, without causing rectal perforation or uncontrolled bleeding. Once adequate luminal patency had been achieved, the next quadrant was approached for trimming. The procedure was completed successfully without any operative or peri-operative complication. Peri-operative flexible sigmoidoscopy confirmed a wide and patent rectal lumen.

## DISCUSSION

Use of the TAMIS approach for ETART to remove a lower-rectal cancer for palliation can be technically very effective, compared with conventional ETART, due to the potential advantages of avoiding contaminant fluid spillage during the procedure, easy access, better visualization than with conventional ETART and being user-friendly. The results from larger cohorts of patients undergoing TAMIS ETART are required before recommending the routine use of this technique Until then, however, this approach may be considered as an alternative to conventional ETART.

**Conflict of interest:** none declared.
